# Can negative results of 40 g glucose load test exclude auto‐brewery syndrome? Comment to: “Auto‐brewery syndrome caused by oral fungi and periodontal disease bacteria” by Takahashi *et al*. Acute Med Surg. 2021 May 3; 8: e652.

**DOI:** 10.1002/ams2.757

**Published:** 2022-05-26

**Authors:** Anna Smędra, Monika Trzmielak, Katarzyna Góralska, Magdalena Dzikowiec, Katarzyna Wochna, Ewa Brzeziańska‐Lasota, Jarosław Berent

**Affiliations:** ^1^ Chair and Department of Forensic Medicine, Medical Faculty Medical University of Lodz Łódź Poland; ^2^ Department of Biology and Parasitology, Chair of Medical Biology and Microbiology, Faculty of Medicine Medical University of Lodz Łódź Poland; ^3^ Department of Biomedicine and Genetics, Chair of Medical Biology and Microbiology, Faculty of Medicine Medical University of Lodz Łódź Poland; ^4^ Department of Criminal Proceedings and Forensics, Faculty of Law and Administration University of Lodz Łódź Poland

Dear Editor,

In 2021 Takahashi *et al*.^1^ published an interesting case report describing a man with auto‐brewery syndrome (ABS), supposedly caused by the production of ethanol by oral fungi and periodontal disease bacteria. The authors reckoned that the production occurred only in the oral cavity and not in subsequent segments of the digestive tract. Their findings were based on *in vitro* tests, which confirmed the production of ethanol by oral microbiota of the patient, and we agree with them. However, the authors further ruled out the possibility of producing ethanol in the intestines, based on the following test: “To rule out alcohol production from the intestinal tract, we thoroughly washed the oral cavity, gave 40 g of glucose to the patient, and measured hourly blood alcohol levels. No blood alcohol levels were detected for 6 h after oral glucose had been given. Therefore, for this patient, alcohol production from the intestinal tract was ruled out as a cause of his seizures.” We disagree with this conclusion.

In our view, the test conducted was not sufficient to exclude the possibility of ethanol production in the intestines because of a number of reasons. According to literature, the test should consist of a 24‐h observation with a high carbohydrate diet and a carbohydrate challenge of 200 g glucose with blood alcohol concentration (BAC) and breath alcohol concentration (BrAC) testing at intervals of 0, 1/2, 1, 2, 4, 8, 16, and 24 h.^2^ The amount of glucose used in the challenge for the reported case was significantly lower, and the duration of the experiment was too short. Ethanol produced because of ABS could have appeared after 6 h, when they stopped BAC testing. Furthermore, the patient had undergone treatment with antifungal and antibacterial drugs before the test, which could have affected gut microbiota and therefore, distorted the results. Yet, the most powerful argument against the findings of Takahashi *et al*. is the fact that the amount of microbiota present in the patient's oral cavity was not sufficient to produce so much ethanol. To reach BAC level over 200 mg/dL, which was found in the patient, ~100 g of pure ethanol needs to be produced. If the patient's intraoral production of ethanol had indeed been so high, it would have caused a strong smell of alcohol on his breath. Takahashi *et al*.^1^ did not observe this symptom, otherwise they would have mentioned it in their publication. Moreover, the photograph included in their publication does not show visible signs of oral candidiasis, or, if any, only insignificant manifestations. Last but not least, ethanol and symptoms caused by its presence should have appeared when their patient consumed food, and they did not. In our opinion, alcohol production in the patient reported by Takahashi *et al*.^1^ occurred throughout the digestive tract, not just in the oral cavity. Naturally, trace amounts of ethanol could have been produced in the oral cavity but it was only a minor addition to a typical intestinal ABS.

In our recent work, we described a case of an oral form of ABS, proving that such phenomenon can occur, but its symptoms are different—this form is characterized by an almost instant production of alcohol in the mouth and its disappearance within minutes.^3^

## DISCLOSURE

Approval of the research protocol with approval No. and committee Name: N/A.

Informed Consent: N/A

Registry and the Registration No. of the study/Trial: N/A

Animal Studies: N/A

Conflict of Interest: None.
